# Psychiatric Admissions and Length of Stay During Fiscal Years 2014 and 2015 in Japan: A Retrospective Cohort Study Using a Nationwide Claims Database

**DOI:** 10.2188/jea.JE20180096

**Published:** 2019-08-05

**Authors:** Yasuyuki Okumura, Naoya Sugiyama, Toshie Noda, Hisateru Tachimori

**Affiliations:** 1 Research Department, Institute for Health Economics and Policy, Association for Health Economics Research and Social Insurance and Welfare, Tokyo, Japan; 2 Department of Psychiatry and Behavioral Science, Tokyo Metropolitan Institute of Medical Science, Tokyo, Japan; 3 Numazu Chuo Hospital, Fukkokai Foundation, Shizuoka, Japan; 4 Atami Chuo Clinic, Fukkokai Foundation, Shizuoka, Japan; 5 National Institute of Mental Health, National Center of Neurology and Psychiatry, Tokyo, Japan

**Keywords:** resource use, claims data, new psychiatric admissions, length of stay, descriptive epidemiology

## Abstract

**Background:**

A better understanding of resource use of new psychiatric admissions is important for healthcare providers and policymakers to improve psychiatric care. This study aims to describe the pattern of new psychiatric admissions and length of stay in Japan.

**Methods:**

A retrospective cohort study was conducted using data from the National Database of Health Insurance Claims and Specific Health Checkups of Japan (NDB). All patients newly admitted to psychiatric wards from April 2014 through March 2016 were included and followed up until discharge to the community.

**Results:**

Our sample included 605,982 admissions from 1,621 hospitals over 2 years. The average monthly number of admissions was 25,024 in fiscal year 2014 and 25,475 in fiscal year 2015. There was a seasonal trend in the number of admissions, with a peak in summer (in July). The discharge rates within 90 days and 360 days were 64.1% and 85.7%, respectively, and varied by type of hospital fee and by hospital. For example, the range of hospital-level discharge rate within 90 days in psychiatric emergency units was 46.0–75.3% in the 1st (lowest) quintile, while it was 83.6–96.0% in the 5th (highest) quintile. The prefecture-level indicators in the NDB and the 630 survey had correlations of >0.70.

**Conclusions:**

Our study provides fundamental information on resource use of new psychiatric admissions in Japan. Although using the NDB has substantial benefits in monitoring resource use, the results should be interpreted with some caution owing to methodological issues inherent in the database.

## INTRODUCTION

The mental health care system in Japan lags behind the trend of deinstitutionalization—the shifting of care from hospitals to communities.^1^ Indeed, the number of psychiatric beds in Japan is four times higher than the Organisation for Economic Co-operation and Development average (267 beds vs 66 beds per 100,000 population).^2^ In 2004, the Japanese government announced a policy for transition from psychiatric inpatient care to community-based mental health care.^3^ Although the length of stay for newly admitted patients shortened after the policy was implemented,^4^ every year 50,000 patients have a long stay in hospital (ie, more than 1 year).^5^ Consequently, in 2014, the government announced a policy to discharge as many newly admitted patients from psychiatric inpatient care within 1 year as possible.^6^

A better understanding of the resources’ use for the new psychiatric admissions is important for healthcare providers and policymakers to improve psychiatric care efficiently.^7^^,^^8^ However, limited information is available on the topic. The “630 survey” is the only nationwide study collecting information on new psychiatric admissions.^9^^,^^10^ Specifically, it is a questionnaire survey of psychiatric hospitals/clinics conducted every year by the Ministry of Health, Labour and Welfare (MHWL).^9^ The 630 survey has several limitations, although its results are extensively used by national and local policymakers. First, the study period is limited to 1 month (ie, new psychiatric admissions occurring in June) rather than a full year. Second, the survey method is not free from non-response biases, although the eligibility criterion is all medical institutions with at least one psychiatric bed. Third, the definition of new psychiatric admissions and their discharge is based on information in a single hospital rather than a single episode of psychiatric admission, which accounts for inter-hospital transfers. Thus, this definition does not identify whether a psychiatric admission is transferred from or discharged to another psychiatric hospital.

Such limitations of the 630 survey can be overcome by using a nationwide claims database—the National Database of Health Insurance Claims and Specific Health Checkups of Japan (NDB). Therefore, we aim to describe the pattern of new psychiatric admissions and length of stay in Japan using the NDB.

## METHODS

### Design and setting

We conducted a retrospective cohort study using data from the NDB. A detailed description of the NDB has been reported elsewhere.^11^ Japan’s population is 127 million, and since April 2009, the MHWL has assembled almost all claims submitted electronically from medical institutions.^12^ The exceptions are medical treatments solely covered by public funds (eg, recipients through the public assistance system) and medical treatments uncovered by public insurance. The NDB includes various information, such as patient identification numbers (ie, the “ID1” generated from the insurance identification number, birth date, and sex, and the “ID2” generated from name, birth date, and sex), hospital codes, prefecture codes, sex, age group, procedural codes, date of procedures, and diagnostic codes. The NDB has been used in several clinical epidemiological studies.^13^^–^^16^ The institutional review board at the Institute of Health Economics and Policy reviewed and approved our study protocol. Due to the anonymous nature of the data, the board waived the requirement for informed consent.

Japan has several types of psychiatric units reimbursed by public health insurance (Table 1). Hospital fees for psychiatric units are classified into three major categories: fee-for-service payment plan, fee-for-service payment plan in advanced treatment hospitals that have ≥400 beds and at least 16 specialties, and per-diem payment plan. There are certification criteria for discharge rates in some per-diem payment plans, including psychiatric emergency units, psychiatric emergency and physical complication units, and psychiatric acute care units (eTable 1). For example, psychiatric acute care units are required to discharge at least 40% of patients to the community within 3 months after admission.

**Table 1.** Characteristics of study population

Characteristic	*n*	%
Route of admission		
Community	516,972	85.3
General ward	89,010	14.7
Type of hospital fee at admission		
Fee-for-service plan (patient-to-nurse ratio)		
Psychiatric unit (10:1)	11,119	1.8
Psychiatric unit (13:1)	30,721	5.1
Psychiatric unit (15:1)	237,973	39.3
Psychiatric unit (18:1)	9,416	1.6
Psychiatric unit (20:1)	2,485	0.4
Specialized psychiatric unit	2,183	0.4
Fee-for-service plan in advanced treatment hospitals (patient-to-nurse ratio)		
Psychiatric unit (7:1)	4,355	0.7
Psychiatric unit (10:1)	7,576	1.3
Psychiatric unit (13:1)	12,668	2.1
Psychiatric unit (15:1)	3,839	0.6
Per-diem payment plan		
Psychiatric emergency unit	69,697	11.5
Psychiatric acute care unit	115,089	19.0
Psychiatric emergency and physical complication unit	3,034	0.5
Child and adolescent psychiatric unit	4,388	0.7
Chronic psychiatric care unit	47,763	7.9
Dementia care unit	43,676	7.2
Type of admission		
Voluntary	390,099	64.4
Involuntary	212,679	35.1
Planned	3,204	0.5
Inter-hospital transfer during episode of psychiatric admissions		
Without	590,164	97.4
With	15,818	2.6
Sex		
Men	267,160	44.1
Women	338,822	55.9
Age, years		
0–19	17,861	2.9
20–39	111,817	18.5
40–64	207,752	34.3
65–74	99,923	16.5
≥75	168,629	27.8
Principal diagnosis (ICD-10 code)		
Organic, including symptomatic, mental disorders (F0)	122,516	20.2
Mental and behavioral disorders due to psychoactive substance use (F1)	37,264	6.1
Schizophrenia, schizotypal and delusional disorders (F2)	205,488	33.9
Mood disorders (F3)	136,074	22.5
Others	104,640	17.3

### Selection of new psychiatric admission

We identified all new admissions to psychiatric wards from April 2014 through March 2016. We followed all patients from January 2013 through September 2016. To trace each patient, we used patient identification numbers called “ID0” that utilize both ID1 and ID2.^17^ We defined a single episode of psychiatric admission as the period from “the date of admission to a psychiatric ward from community settings (ie, home or institution) or general ward” to “the date of hospital discharge to community settings, general ward, or death”. We deemed patients transferred from one psychiatric ward to other types of psychiatric wards and patients transferred from one psychiatric hospital to another as a single episode. To focus only on new admissions, we excluded those hospitalized in a psychiatric ward before April 1, 2014 and staying in the ward on April 1, 2014. To confirm new admissions, we included patients who enrolled in the database at least 1 day before the admission date. The enrollment status in the database was verified by the existence of any medical claims. We included all new psychiatric admissions with two or more admissions during the study period. We excluded patients hospitalized in two or more hospitals on the same day owing to identification code errors.

### Outcomes

The primary outcomes were the number of new psychiatric admissions and time to hospital discharge to the community. Discharges to the general ward and death within a psychiatric ward were treated as competing risks. As a censoring point for all admissions still without hospital discharge, we used September 30, 2016 as the date and 365 days after admission.

### Other variables

For each episode of psychiatric admission, we extracted information on hospital location based on prefecture codes. We also extracted patient characteristics, such as type of hospital fee at admission, type of admission (involuntary, voluntary, or planned admission), inter-hospital transfer during the psychiatric admission episode, sex (men or women), age group (0–19, 20–39, 40–64, 65–74, or ≥75 years), and principle diagnosis at admission (ICD-10 codes: F0, F1, F2, F3, or others). We defined planned admission as the use of electroconvulsive therapy and length of stay ≤3 days. For each admission, one principle diagnosis was selected based on the MHLW’s algorism.^18^

### Statistical analyses

First, we described the number of psychiatric admissions using the characteristics listed in Table 1. Second, we used a generalized additive model to examine the trend in the monthly number of new psychiatric admissions over 2 years.^19^ Third, we estimated the cumulative incidence of discharge using the Aalen-Johansen estimator, which can account for competing risks.^20^ We also estimated the cumulative incidence of discharge to the community by type of hospital fee. Fourth, for each hospital fee, we estimated the hospital-level discharge rates within 90 days from admission. We excluded hospitals in units with <10 patients due to uncertainty in estimates. The hospital-level discharge rates by hospital fee were sorted in ascending order and grouped into five categories for units with ≥20 hospitals, and into three categories for units with <20 hospitals. Fifth, we compared the following prefecture-level indicators using data from the NDB and the 630 survey in 2015 (ie, new admissions during June 2014).^9^ From the 630 survey, we extracted information on the number of (1) new psychiatric admissions by prefecture, (2) new psychiatric admissions by prefecture and age group, (3) new psychiatric admissions by prefecture and diagnostic category, and (4) the proportion of hospital discharge to community within 1 year by prefecture. We also computed the above-mentioned indicators using the NDB for fiscal year 2014–2015, 2014, and 2015, respectively. The prefecture-level indicators from the NDB and the 630 surveys were compared using Pearson correlation coefficients. All statistical analyses were performed using R version 3.4.1 (R Foundation for Statistical Computing, Vienna, Austria) with the riskRegression package for the competing risk model.^21^ The significance level was set at 5%.

## RESULTS

### Descriptive characteristics

Our sample included 605,982 new psychiatric admissions from 1,621 hospitals over 2 years (eFigure 1). Of these, 85.3% resided in community, 39.3% were reimbursed by hospitalization fee called psychiatric units (15:1), 64.4% were voluntary admissions, and 2.6% were transferred to other psychiatric hospitals during the episodes. The male:female ratio was 0.8:1, and the largest age group was 40–64 years (34.3% of admissions). The most common principle diagnosis was schizophrenia (F2) (33.9%), followed by mood disorders (F3) (22.5%), and organic mental disorders (F0) (20.2%).

### Seasonal trends of new psychiatric admissions

**Figure 1.**
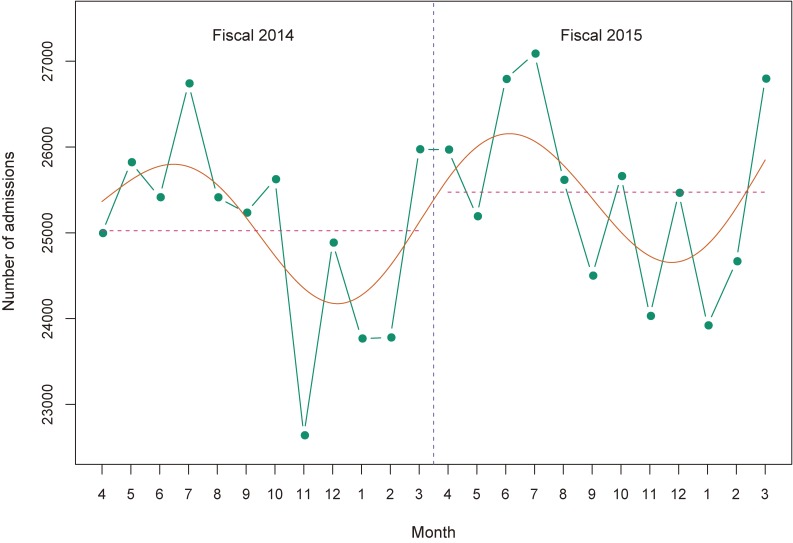
Number of new psychiatric admissions by month.

The average monthly number of new psychiatric admissions was 25,024 in fiscal 2014 and 25,475 in fiscal 2015, respectively. Figure 1 shows that seasonal rather than monotonic trends exist in the number of admissions, which increased in summer, peaked in July, and decreased in winter.

### Patient-level discharge rate

**Figure 2.**
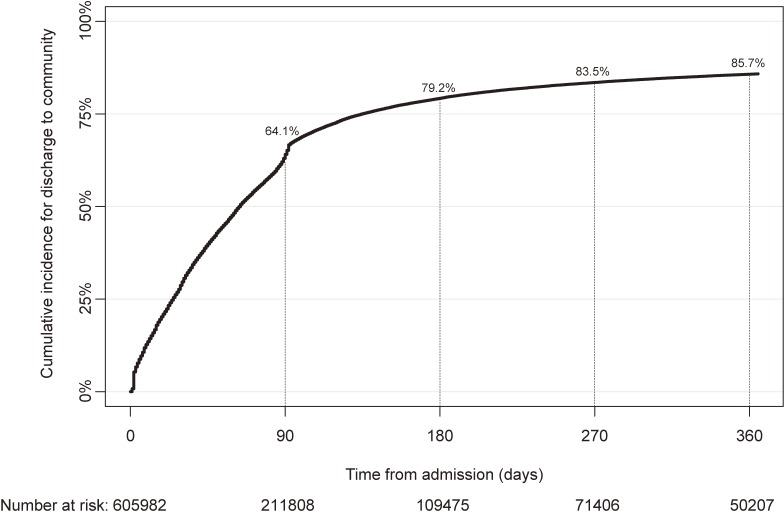
Cumulative incidence of discharge to community.

The patient-level discharge rates for the community were 64.1% at 90 days and 85.7% at 360 days, respectively (Figure 2). The competing event rates at 360 days were 0.3% for transfer to general wards and 3.1% for death. The discharge rates for community varied by type of hospital fee (Table 2, eFigure 2, eFigure 3, and eFigure 4). For example, the discharge rate within 360 days was 98.7% in psychiatric units (7:1) in advanced treatment hospitals, but only 68.0% in dementia care units (Table 2). In psychiatric emergency units, the cumulative incidence of discharge increased steeply until around 90 days from admission, and then the incidence increased gradually in the subsequent period (eFigure 4). Such a piecewise (segmented) trend was also observed in psychiatric acute care units (eFigure 4).

**Table 2.** Cumulative incidence of discharge to community by type of hospital fee

Type of hospital fee at admission	Number of participants	Cumulative incidence of discharge by time from admission, %			

		90 days	180 days	270 days	360 days
Fee-for-service plan (patient-to-nurse ratio)					
Psychiatric unit (10:1)	11,119	86.2	92.2	93.7	94.3
Psychiatric unit (13:1)	30,721	74.3	88.2	91.4	92.7
Psychiatric unit (15:1)	237,973	57.2	73.5	78.7	81.4
Psychiatric unit (18:1)	9,416	53.8	67.8	73.5	76.2
Psychiatric unit (20:1)	2,485	52.0	72.7	79.1	81.8
Specialized psychiatric unit	2,183	47.9	65.6	71.1	74.4
Fee-for-service plan in advanced treatment hospitals (patient-to-nurse ratio)					
Psychiatric unit (7:1)	4,355	93.8	97.5	98.4	98.7
Psychiatric unit (10:1)	7,576	90.6	96.5	97.8	98.1
Psychiatric unit (13:1)	12,668	79.8	93.7	96.2	97.1
Psychiatric unit (15:1)	3,839	75.9	91.6	95.2	96.9
Per-diem payment plan					
Psychiatric emergency unit	69,697	79.2	92.5	94.9	96.0
Psychiatric acute care unit	115,089	75.1	91.0	93.7	94.8
Psychiatric emergency and physical complication unit	3,034	80.2	92.1	94.8	95.7
Child and adolescent psychiatric unit	4,388	59.9	84.6	92.6	95.9
Chronic psychiatric care unit	47,763	52.2	65.6	70.9	74.1
Dementia care unit	43,676	38.3	55.7	63.4	68.0

### Hospital-level discharge rate

The discharge rates within 90 days varied by hospital (Table 3). For example, the range of hospital-level discharge rate in psychiatric emergency units was 46.0–75.3% in the 1st (lowest) discharge rate quintile, while it was 83.6–96.0% in the 5th (highest) quintile (Table 3). The range of hospital-level discharge rate in dementia care units was 0.0–23.4% in the 1st quintile, while it was 47.7–87.7% in the 5th quintile.

**Table 3.** Hospital-level discharge rates within 90 days from admissions by type of hospital fee

Type of hospital fee at admission	Number of hospitals	Number of hospitals with ≥10 patients	Hospital-level discharge rates within 90-day by hospital ranking (3 or 5 groups), range				

			1st (lowest)	2nd	3rd	4th	5th (highest)
Fee-for-service plan (patient-to-nurse ratio)							
Psychiatric unit (10:1)	23	23	69.9–77.6	77.7–81.1	81.2–87.6	87.7–91.0	91.1–99.5
Psychiatric unit (13:1)	97	95	39.4–66.0	66.1–73.9	74.0–78.3	78.4–83.3	83.4–96.0
Psychiatric unit (15:1)	1,272	1,250	3.6–42.4	42.5–51.1	51.2–58.3	58.4–66.3	66.4–100.0
Psychiatric unit (18:1)	75	68	10.0–34.1	34.2–45.3	45.4–56.6	56.7–64.4	64.5–80.0
Psychiatric unit (20:1)	22	18	20.7–35.6	—	35.7–51.0	—	51.1–78.0
Specialized psychiatric unit	50	30	0.0–33.9	34.0–46.6	46.7–56.0	56.1–61.2	61.3–85.4
Fee-for-service plan in advanced treatment hospitals (patient-to-nurse ratio)							
Psychiatric unit (7:1)	10	10	87.4–91.5	—	91.6–94.6	—	94.7–97.0
Psychiatric unit (10:1)	15	15	82.4–89.9	—	90.0–92.9	—	93.0–97.0
Psychiatric unit (13:1)	40	40	68.7–72.6	72.7–77.6	77.7–80.7	80.8–85.0	85.1–91.0
Psychiatric unit (15:1)	20	19	60.4–70.4	—	70.5–77.8	—	77.9–90.0
Per-diem payment plan							
Psychiatric emergency unit	131	131	46.0–75.3	75.4–77.9	78.0–80.8	80.9–83.5	83.6–96.0
Psychiatric acute care unit	370	369	42.8–67.8	67.9–73.1	73.2–77.8	77.9–81.2	81.3–95.9
Psychiatric emergency and physical complication unit	11	11	68.3–79.2	—	79.3–82.8	—	82.9–92.0
Child and adolescent psychiatric unit	36	35	31.6–42.5	42.6–52.1	52.2–61.4	61.5–72.6	72.7–84.2
Chronic psychiatric care unit	841	724	0.0–25.6	25.7–39.9	40.0–49.9	50.0–60.7	60.8–94.8
Dementia care unit	515	486	0.0–23.4	23.5–32.2	32.3–38.6	38.7–47.6	47.7–87.7

### Concordance of prefecture-level indicators between the NDB and the 630 survey

The number of new psychiatric admissions during June 2014 was lower in the NDB than in the 630 survey (25,414 vs 31,669) (Figure 1). The discharge rate within 1 year was higher in the NDB than in the 630 survey (85.7% vs 74.1%) (Figure 2). The prefecture-level indicators for the number of new psychiatric admissions in the NDB and the 630 survey had high correlations of >0.95 (Table 4). However, the prefecture-level discharge rates within 1 year in the NDB and the 630 survey had moderate correlations of >0.70 (Table 4). The prefecture-level indicators from the NDB are available in the online appendix (eTable 2, eTable 3, eTable 4, and eTable 5).

**Table 4.** Pearson correlation coefficients for 4 indicators using data from the NDB and the 630 survey

Indicator	Admission year in data from the NDB, fiscal year		

	2014–2015	2014	2015
1. All	0.981^*^	0.981^*^	0.980^*^
2. Age group, year			
0–19	0.968^*^	0.971^*^	0.961^*^
20–39	0.974^*^	0.976^*^	0.971^*^
40–64	0.977^*^	0.977^*^	0.977^*^
65–74	0.957^*^	0.959^*^	0.953^*^
≥75	0.986^*^	0.984^*^	0.986^*^
3. Diagnostic category (ICD-10 code)			
Organic, including symptomatic, mental disorders (F0)	0.978^*^	0.978^*^	0.977^*^
Mental and behavioral disorders due to psychoactive substance use (F1)	0.960^*^	0.964^*^	0.950^*^
Schizophrenia, schizotypal and delusional disorders (F2)	0.969^*^	0.967^*^	0.969^*^
Mood disorders (F3)	0.981^*^	0.982^*^	0.979^*^
Others	0.964^*^	0.961^*^	0.965^*^
4. Proportion of discharge to community at 1 year	0.755^*^	0.762^*^	0.701^*^

NDB, National Database of Health Insurance Claims and Specific Health Checkups of Japan.

^*^P < 0.05.

## DISCUSSION

This is the first study in Japan to describe a pattern of new psychiatric admissions and length of stay using the nationwide claims database. We found that the discharge rate within 360 days was 86% in the entire population, varying by types of units (range: 68% to 99%). We do note that the differences in the discharge rate by types of units are strongly influenced by the differences in the patient characteristics by types of units. For example, patients with first-episode schizophrenia are more likely to be admitted to psychiatric units with some certification criteria for length of stay than those with chronic schizophrenia, because of the expectation that patients with first-episode schizophrenia commonly require shorter lengths of stay. However, the government has advocated that psychiatric care systems should have the capability to discharge newly admitted psychiatric patients within a year, if possible.^6^ Given the large difference in the discharge rate by types of units, more realistic goals and effective plans would be required by the type of units.

We also observed piecewise trends in the cumulative incidence for discharge in psychiatric emergency units and acute psychiatric units. The trend of discharge until around 90 days after admissions was much steeper than in the subsequent period. This may be due to the effect of the certification requirements for discharge rate. However, it remains unknown whether such unnatural trend is a reflection of the negative effects of the certification criteria. Future studies should assess the rationale for piecewise trends.

Our findings have significant implications for the efficient improvement of psychiatric care by healthcare providers and policymakers. For example, healthcare providers could understand their current practices from our results on hospital-level discharge rates by type of hospital fee. This awareness would contribute to setting a realistic goal for quality improvement.^22^ Moreover, local governments could monitor the effectiveness of their policies in supporting discharge from hospital to community, using our findings on prefecture-level discharge rates. Furthermore, national and local policymakers can use our estimates as the basis for estimating future medical needs for new psychiatric admissions.^23^

Our study has the advantages of having a representative study period, being free of non-response biases, and having a valid definition of new psychiatric admissions. A comparison with the NDB and the 630 survey gives insights into the underlying nature of statistics. First, the number of new psychiatric admissions in Japan was around 25,000 per month, which was 20% lower than that in the 630 survey. A major reason for this is that our data did not include the claims solely covered by public funds.^24^ This may result in a 19% underestimation.^25^ Another possible reason is that our definition of new psychiatric admissions is based on a single episode, which includes inter-hospital transfers. However, this possible reason is not a problem per se, as our definition may lead to more realistic estimates of new psychiatric admissions.

Second, the rate of discharge to the community within 360 days was 85%, which was 15% higher than that in the 630 survey. Here too, a possible reason is that our data did not include the claims solely covered by public funds.^24^ For example, patients with public assistance might be more likely to experience prolonged hospitalization than those without. This may result in overestimation of the discharge rate. Another possible reason is that our definition of discharge to the community is based on a single episode considering inter-hospital transfers. This definition may contribute to more accurate estimates of discharge to the community. On the contrary, the definition in the 630 survey would lead to underestimation due to missing follow-up data on patients transferred to other psychiatric hospitals.

Third, through a visual inspection of the graph, we found a seasonal pattern in the number of psychiatric admissions with a peak in summer (in July). Our findings are consistent with those of previous studies conducted in Italy and Vietnam.^26^^,^^27^ However, our findings of the seasonal trends are preliminary evidence due to the short-term observational period. Future studies should confirm the existence of seasonal trends to assess the magnitude of bias in the annual number of psychiatric admissions, based on data from a single month.

Our study has several limitations. First, our data did not include the claims solely covered by public funds.^24^ This results in underestimation of the number of psychiatric admissions and probably overestimation of the discharge rate. Future studies should assess the magnitude of bias in the discharge rate. Second, our study period was limited to only 2 fiscal years. Thus, studies with longer study periods will be necessary to confirm the potential seasonal effects of the number of psychiatric admissions. Third, the occurrence of death in our study can result in misclassification bias. Although the death rate in our study (3.1%) was quite similar to that in the 630 survey (3.3%),^9^ the magnitude of this bias is not known in psychiatric settings. Fourth, the unit of our analyses was a psychiatric admission rather a patient, which made it difficult for us to describe the readmission rate. Further studies that consider the patient as the unit of analysis should focus on better understanding the resource use of new psychiatric admissions.

### Conclusions

Our study provides fundamental information on resource use for new psychiatric admissions in terms of the number of admissions and length of stay in Japan. National and local policymakers can develop a better understanding of resource use and estimate future medical needs for new psychiatric admissions. Our findings would also be beneficial in enabling healthcare providers and policymakers to know hospital- and prefecture-level discharge rates. Although using the nationwide claims database is beneficial in monitoring resource use, the results should be interpreted with some caution due to methodological issues inherent in the database.

## APPENDIX A. SUPPLEMENTARY DATA

eFigure 1. Flow diagram of included participants

eFigure 2. Cumulative incidence of discharge to community by type of hospital fee (fee-for-service plan)

eFigure 3. Cumulative incidence of discharge to community by type of hospital fee (fee-for-service plan in advanced treatment hospitals)

eFigure 4. Cumulative incidence of discharge to community by type of hospital fee (per-diem payment plan)

eTable 1. Target population and major certification criteria among psychiatric units

eTable 2. Number of new psychiatric admissions by prefecture

eTable 3. Number of new psychiatric admissions by prefecture and age group

eTable 4. Number of new psychiatric admissions by prefecture and diagnostic category

eTable 5. Percentage of discharge to community within 360 days after discharge by prefecture
