# Supplementary material for: Psychiatric Admissions and Length of Stay During Fiscal Years 2014 and 2015 in Japan: A Retrospective Cohort Study Using a Nationwide Claims Database
Source: J Epidemiol. 2019 Aug 5;29(8):288–94. doi: 10.2188/jea.JE20180096 (PMC6614081; doi:10.2188/jea.JE20180096)
Supplement: Supplementary file 1 [file je-29-288-s001.pdf]

eTable 1. Target population and major certification criteria among psychiatric units

Type of hospital fee	Target population	Major certification requirement
Fee-for-service plan (patient-to-nurse ratio)		
Psychiatric unit (10:1)	Patients with mental disorders	-Average length of stay ≤40 days in the unit<br>-Among newly admitted patients in the unit, at least 50% with severe impairment (GAF score ≤30) at admission
Psychiatric unit (13:1)	Patients with mental disorders	-Average length of stay ≤80 days in the unit<br>-Among newly admitted patients in the unit, at least 40% with severe impairment (GAF score ≤30) or comorbid with physical illness at admission
Psychiatric unit (15:1)	Patients with mental disorders	—
Psychiatric unit (18:1)	Patients with mental disorders	—
Psychiatric unit (20:1)	Patients with mental disorders	—
Specialized psychiatric unit (25:1)	Patients with mental disorders	—
Fee-for-service plan in advanced treatment hospitals (patient-to-nurse ratio)		
Psychiatric unit (7:1)	Patients with mental disorders	-Average length of stay ≤40 days in the unit<br>-Among newly admitted patients in the unit, at least 50% with severe impairment (GAF score ≤30) at admission
Psychiatric unit (10:1)	Patients with mental disorders	-Average length of stay ≤40 days in the unit<br>-Among newly admitted patients in the unit, at least 50% with severe impairment (GAF score ≤30) at admission
Psychiatric unit (13:1)	Patients with mental disorders	-Average length of stay ≤80 days in the unit<br>-Among newly admitted patients in the unit, at least 40% with severe impairment (GAF score ≤30) or comorbid with physical illness at admission
Psychiatric unit (15:1)	Patients with mental disorders	—
Per-diem payment plan		
Psychiatric emergency unit	Patients who were involuntarily admitted or who have no history of psychiatric admission for at least 3 months	-Among newly admitted patients in the unit, at least 60% (or 40% in the units reimbursed by lower hospital fee) discharged to community within 3 months
Psychiatric acute care unit	Patients who have no history of psychiatric admission for at least 3 months or who require inter-hospital transfer due to acute exacerbation	-Among newly admitted patients in the unit, at least 40% (or 60% in units with patient-to-psychiatrist ratio of 16:1) discharged to community within 3 months
Psychiatric emergency and physical complication unit	Patients who were involuntarily admitted, who have no history of psychiatric admission for at least 3 months, or who require intensive care for physical illness during admission	-Among newly admitted patients in the unit, at least 40% discharged to community within 3 months<br>-Among newly admitted patients in the unit, at least 60% admitted involuntarily or comorbid with physical illness
Child and adolescent psychiatric unit	Patients with mental disorders aged 0–19 years	-Among patients hospitalized in the unit, approximately at least 80% aged 0–19 years
Chronic psychiatric care unit	Patients with mental disorders who require long-term care	—
Dementia care unit	Patients with dementia who require intensive care for severe behavioral and psychological symptoms	—

GAF, global assessment of functioning.

eTable 2. Number of new psychiatric admissions by prefecture

	Fiscal year		
Prefecture	2014–2015	2014	2015
Hokkaido	34,114	16,686	17,428
Aomori	9,386	4,679	4,707
Iwate	8,417	4,346	4,071
Miyagi	10,763	5,279	5,484
Akita	7,984	4,049	3,935
Yamagata	9,525	4,565	4,960
Fukushima	10,407	5,274	5,133
Ibaraki	11,940	6,100	5,840
Tochigi	7,805	3,815	3,990
Gunma	9,452	4,712	4,740
Saitama	26,876	13,225	13,651
Chiba	18,272	9,107	9,165
Tokyo	50,076	24,506	25,570
Kanagawa	29,265	14,427	14,838
Niigata	11,176	5,441	5,735
Toyama	6,274	3,109	3,165
Ishikawa	8,414	4,078	4,336
Fukui	5,377	2,665	2,712
Yamanashi	4,669	2,395	2,274
Nagano	12,010	5,909	6,101
Gifu	9,417	4,725	4,692
Shizuoka	13,380	6,593	6,787
Aichi	28,329	14,023	14,306
Mie	8,344	4,182	4,162
Shiga	4,854	2,431	2,423
Kyoto	10,533	5,085	5,448
Osaka	33,447	16,778	16,669
Hyogo	20,678	10,107	10,571
Nara	5,499	2,720	2,779
Wakayama	3,013	1,480	1,533
Tottori	3,517	1,735	1,782
Shimane	5,286	2,563	2,723
Okayama	11,391	5,738	5,653
Hiroshima	16,121	8,007	8,114
Yamaguchi	8,761	4,514	4,247
Tokushima	4,697	2,284	2,413
Kagawa	5,386	2,699	2,687
Ehime	7,372	3,675	3,697
Kochi	6,422	3,208	3,214
Fukuoka	33,361	16,401	16,960
Saga	8,093	4,060	4,033
Nagasaki	10,461	5,283	5,178
Kumamoto	15,970	7,925	8,045
Oita	7,296	3,722	3,574
Miyazaki	9,133	4,519	4,614
Kagoshima	13,263	6,524	6,739
Okinawa	9,756	4,939	4,817

eTable 3. Number of new psychiatric admissions by prefecture and age group

	Fiscal year 2014–2015					Fiscal year 2014					Fiscal year 2015				
	Age group, years					Age group, years					Age group, years				
Prefecture	0–19	20–39	40–64	65–74	≥75	0–19	20–39	40–64	65–74	≥75	0–19	20–39	40–64	65–74	≥75
Hokkaido	883	5,537	10,123	5,466	12,105	415	2,744	4,989	2,623	5,915	468	2,793	5,134	2,843	6,190
Aomori	335	1,598	2,769	1,551	3,133	152	813	1,455	700	1,559	183	785	1,314	851	1,574
Iwate	262	1,819	2,913	1,305	2,118	123	1,011	1,444	699	1,069	139	808	1,469	606	1,049
Miyagi	408	2,085	3,788	1,591	2,891	198	1,047	1,755	875	1,404	210	1,038	2,033	716	1,487
Akita	160	1,190	2,479	1,260	2,895	95	640	1,268	616	1,430	65	550	1,211	644	1,465
Yamagata	305	1,466	3,034	1,527	3,193	158	687	1,546	657	1,517	147	779	1,488	870	1,676
Fukushima	286	1,968	3,506	1,721	2,926	146	1,064	1,803	813	1,448	140	904	1,703	908	1,478
Ibaraki	356	2,330	4,277	1,751	3,226	178	1,162	2,258	875	1,627	178	1,168	2,019	876	1,599
Tochigi	312	1,368	2,974	1,443	1,708	171	706	1,467	675	796	141	662	1,507	768	912
Gunma	219	1,820	3,716	1,832	1,865	97	887	1,842	977	909	122	933	1,874	855	956
Saitama	541	4,843	8,687	4,714	8,091	263	2,453	4,396	2,257	3,856	278	2,390	4,291	2,457	4,235
Chiba	539	4,031	7,165	3,127	3,410	252	2,077	3,544	1,578	1,656	287	1,954	3,621	1,549	1,754
Tokyo	2,097	11,552	18,275	7,159	10,993	977	5,820	8,973	3,577	5,159	1,120	5,732	9,302	3,582	5,834
Kanagawa	1,241	5,890	10,680	4,517	6,937	634	2,952	5,363	2,166	3,312	607	2,938	5,317	2,351	3,625
Niigata	380	1,912	3,652	1,904	3,328	176	915	1,735	946	1,669	204	997	1,917	958	1,659
Toyama	141	1,048	2,175	1,095	1,815	73	533	1,081	554	868	68	515	1,094	541	947
Ishikawa	163	1,568	2,743	1,503	2,437	69	756	1,322	734	1,197	94	812	1,421	769	1,240
Fukui	147	912	1,655	838	1,825	65	495	825	397	883	82	417	830	441	942
Yamanashi	162	874	1,797	792	1,044	82	431	929	421	532	80	443	868	371	512
Nagano	515	2,386	4,547	2,068	2,494	232	1,175	2,306	999	1,197	283	1,211	2,241	1,069	1,297
Gifu	208	1,908	3,503	1,547	2,251	95	967	1,797	784	1,082	113	941	1,706	763	1,169
Shizuoka	715	2,646	4,891	2,145	2,983	363	1,364	2,343	1,056	1,467	352	1,282	2,548	1,089	1,516
Aichi	925	6,151	10,750	5,446	5,057	422	3,126	5,403	2,625	2,447	503	3,025	5,347	2,821	2,610
Mie	230	1,687	3,087	1,320	2,020	107	841	1,596	653	985	123	846	1,491	667	1,035
Shiga	184	1,045	1,651	793	1,181	81	529	838	420	563	103	516	813	373	618
Kyoto	167	1,704	3,492	1,852	3,318	89	878	1,654	873	1,591	78	826	1,838	979	1,727
Osaka	1,022	6,165	11,716	5,854	8,690	530	3,182	5,940	2,864	4,262	492	2,983	5,776	2,990	4,428
Hyogo	596	3,719	7,009	3,352	6,002	292	1,840	3,458	1,696	2,821	304	1,879	3,551	1,656	3,181
Nara	107	984	1,771	914	1,723	48	496	888	456	832	59	488	883	458	891
Wakayama	43	698	1,201	541	530	18	337	592	275	258	25	361	609	266	272
Tottori	36	654	1,147	559	1,121	18	320	570	273	554	18	334	577	286	567
Shimane	139	868	1,711	920	1,648	65	422	843	450	783	74	446	868	470	865
Okayama	376	1,900	3,631	2,044	3,440	158	1,013	1,817	1,037	1,713	218	887	1,814	1,007	1,727
Hiroshima	461	2,627	5,022	2,846	5,165	222	1,421	2,561	1,337	2,466	239	1,206	2,461	1,509	2,699
Yamaguchi	145	1,268	2,937	1,469	2,942	69	694	1,537	752	1,462	76	574	1,400	717	1,480
Tokushima	85	783	1,928	873	1,028	43	376	960	444	461	42	407	968	429	567
Kagawa	205	874	1,726	894	1,687	100	455	866	476	802	105	419	860	418	885
Ehime	135	1,152	2,670	1,259	2,156	66	559	1,356	627	1,067	69	593	1,314	632	1,089
Kochi	157	775	1,818	1,147	2,525	59	372	946	557	1,274	98	403	872	590	1,251
Fukuoka	607	5,766	10,592	5,724	10,672	304	2,855	5,283	2,779	5,180	303	2,911	5,309	2,945	5,492
Saga	287	1,417	2,838	1,339	2,212	146	707	1,420	700	1,087	141	710	1,418	639	1,125
Nagasaki	296	1,764	3,494	1,598	3,309	146	921	1,761	772	1,683	150	843	1,733	826	1,626
Kumamoto	615	2,634	5,038	2,531	5,152	292	1,289	2,577	1,247	2,520	323	1,345	2,461	1,284	2,632
Oita	135	1,019	2,185	1,172	2,785	60	565	1,173	545	1,379	75	454	1,012	627	1,406
Miyazaki	179	1,267	2,755	1,398	3,534	95	628	1,366	669	1,761	84	639	1,389	729	1,773
Kagoshima	189	2,173	4,126	1,977	4,798	105	1,030	2,066	963	2,360	84	1,143	2,060	1,014	2,438
Okinawa	165	1,972	4,108	1,245	2,266	88	1,003	2,189	561	1,098	77	969	1,919	684	1,168

eTable 4. Number of new psychiatric admissions by prefecture and diagnostic category

	Fiscal year 2014–2015					Fiscal year 2014					Fiscal year 2015				
Prefecture	F0	F1	F2	F3	Others	F0	F1	F2	F3	Others	F0	F1	F2	F3	Others
Hokkaido	8,510	1,853	9,226	8,296	6,229	4,142	901	4,454	4,076	3,113	4,368	952	4,772	4,220	3,116
Aomori	2,693	594	2,737	1,859	1,503	1,277	296	1,398	920	788	1,416	298	1,339	939	715
Iwate	1,539	500	3,336	1,776	1,266	773	260	1,780	904	629	766	240	1,556	872	637
Miyagi	2,266	710	3,458	2,189	2,140	1,070	353	1,693	1,145	1,018	1,196	357	1,765	1,044	1,122
Akita	1,827	285	1,898	1,484	2,490	885	131	966	747	1,320	942	154	932	737	1,170
Yamagata	1,969	406	2,797	2,179	2,174	897	168	1,365	1,032	1,103	1,072	238	1,432	1,147	1,071
Fukushima	2,200	439	3,729	2,448	1,591	1,108	215	1,868	1,315	768	1,092	224	1,861	1,133	823
Ibaraki	1,975	494	5,291	2,235	1,945	973	262	2,721	1,134	1,010	1,002	232	2,570	1,101	935
Tochigi	1,004	223	2,971	2,106	1,501	474	108	1,489	989	755	530	115	1,482	1,117	746
Gunma	1,284	872	4,158	1,688	1,450	619	410	2,183	804	696	665	462	1,975	884	754
Saitama	6,817	1,053	9,199	6,033	3,774	3,326	514	4,708	2,878	1,799	3,491	539	4,491	3,155	1,975
Chiba	2,288	1,244	7,717	4,712	2,311	1,104	632	3,851	2,405	1,115	1,184	612	3,866	2,307	1,196
Tokyo	7,042	3,217	17,208	13,087	9,522	3,361	1,513	8,498	6,515	4,619	3,681	1,704	8,710	6,572	4,903
Kanagawa	5,210	2,086	11,376	6,630	3,963	2,406	1,064	5,599	3,245	2,113	2,804	1,022	5,777	3,385	1,850
Niigata	2,437	463	3,733	2,495	2,048	1,194	205	1,755	1,251	1,036	1,243	258	1,978	1,244	1,012
Toyama	1,318	208	2,129	1,485	1,134	674	95	1,029	748	563	644	113	1,100	737	571
Ishikawa	2,040	325	2,569	1,943	1,537	1,005	158	1,217	982	716	1,035	167	1,352	961	821
Fukui	1,430	182	1,302	1,264	1,199	687	94	635	613	636	743	88	667	651	563
Yamanashi	592	331	1,967	964	815	328	147	1,023	500	397	264	184	944	464	418
Nagano	1,337	732	4,223	2,785	2,933	621	366	2,163	1,336	1,423	716	366	2,060	1,449	1,510
Gifu	1,551	642	3,338	2,327	1,559	755	308	1,720	1,156	786	796	334	1,618	1,171	773
Shizuoka	1,916	967	5,069	3,079	2,349	953	497	2,428	1,524	1,191	963	470	2,641	1,555	1,158
Aichi	3,962	1,214	10,518	7,353	5,282	1,850	607	5,291	3,683	2,592	2,112	607	5,227	3,670	2,690
Mie	1,545	482	3,041	1,665	1,611	744	244	1,532	838	824	801	238	1,509	827	787
Shiga	826	221	1,594	1,516	697	398	115	828	756	334	428	106	766	760	363
Kyoto	2,718	602	3,692	2,249	1,272	1,325	272	1,778	1,060	650	1,393	330	1,914	1,189	622
Osaka	6,835	2,878	11,182	7,629	4,923	3,277	1,521	5,655	3,854	2,471	3,558	1,357	5,527	3,775	2,452
Hyogo	4,891	1,149	7,440	3,664	3,534	2,340	541	3,646	1,802	1,778	2,551	608	3,794	1,862	1,756
Nara	1,375	118	1,969	1,271	766	652	64	979	643	382	723	54	990	628	384
Wakayama	309	85	1,358	710	551	146	41	681	347	265	163	44	677	363	286
Tottori	901	227	982	783	624	428	123	487	385	312	473	104	495	398	312
Shimane	1,050	298	1,575	1,079	1,284	486	148	751	529	649	564	150	824	550	635
Okayama	2,456	909	3,535	2,480	2,011	1,211	428	1,769	1,301	1,029	1,245	481	1,766	1,179	982
Hiroshima	3,803	1,461	4,738	3,389	2,730	1,876	620	2,425	1,712	1,374	1,927	841	2,313	1,677	1,356
Yamaguchi	2,192	657	2,919	1,567	1,426	1,144	321	1,513	811	725	1,048	336	1,406	756	701
Tokushima	821	334	1,781	914	847	400	161	901	432	390	421	173	880	482	457
Kagawa	759	402	1,777	868	1,580	364	193	912	443	787	395	209	865	425	793
Ehime	1,694	448	2,754	1,397	1,079	858	227	1,364	700	526	836	221	1,390	697	553
Kochi	1,795	590	1,709	1,113	1,215	942	304	883	531	548	853	286	826	582	667
Fukuoka	8,193	2,148	9,639	8,221	5,160	3,928	1,080	4,888	4,010	2,495	4,265	1,068	4,751	4,211	2,665
Saga	1,468	651	2,299	2,015	1,660	717	336	1,072	1,044	891	751	315	1,227	971	769
Nagasaki	2,145	707	3,014	2,473	2,122	1,094	351	1,493	1,239	1,106	1,051	356	1,521	1,234	1,016
Kumamoto	3,550	946	4,767	3,987	2,720	1,768	478	2,404	1,950	1,325	1,782	468	2,363	2,037	1,395
Oita	1,946	514	2,406	1,421	1,009	918	251	1,291	706	556	1,028	263	1,115	715	453
Miyazaki	2,632	665	2,489	1,627	1,720	1,332	339	1,168	842	838	1,300	326	1,321	785	882
Kagoshima	3,386	921	4,472	2,215	2,269	1,636	481	2,256	1,100	1,051	1,750	440	2,216	1,115	1,218
Okinawa	2,019	811	4,407	1,404	1,115	987	420	2,287	710	535	1,032	391	2,120	694	580

eTable 5. Percentage of discharge to community within 360 days after discharge by prefecture

	Fiscal year		
Prefecture	2014–2015	2014	2015
Hokkaido	84.8	84.8	84.9
Aomori	87.9	87.6	88.4
Iwate	85.4	85.3	85.6
Miyagi	84.1	83.5	84.7
Akita	85.2	84.4	86.2
Yamagata	86.2	87.0	85.4
Fukushima	84.8	84.6	84.8
Ibaraki	83.4	83.7	82.9
Tochigi	86.0	86.5	85.5
Gunma	85.2	84.9	85.5
Saitama	83.7	84.0	83.4
Chiba	86.7	86.2	87.3
Tokyo	90.5	90.5	90.5
Kanagawa	88.0	88.6	87.3
Niigata	82.9	82.2	83.7
Toyama	84.2	83.0	85.4
Ishikawa	87.5	86.7	88.4
Fukui	87.3	87.3	87.3
Yamanashi	88.6	87.8	89.6
Nagano	87.4	86.5	88.3
Gifu	87.3	87.0	87.5
Shizuoka	88.4	88.4	88.5
Aichi	89.2	88.7	89.6
Mie	85.8	85.2	86.5
Shiga	85.6	84.8	86.7
Kyoto	83.8	83.6	83.9
Osaka	87.8	87.2	88.4
Hyogo	85.9	85.4	86.4
Nara	86.5	85.8	87.2
Wakayama	88.8	89.5	88.1
Tottori	84.8	83.1	86.7
Shimane	83.4	83.4	83.5
Okayama	86.1	86.1	86.3
Hiroshima	83.0	84.2	81.6
Yamaguchi	78.0	78.4	77.6
Tokushima	85.5	83.6	87.4
Kagawa	82.3	82.7	81.9
Ehime	85.0	84.6	85.6
Kochi	87.0	86.4	87.6
Fukuoka	84.2	83.5	84.8
Saga	83.8	83.3	84.1
Nagasaki	82.0	81.4	82.6
Kumamoto	85.6	85.7	85.5
Oita	80.9	81.8	80.0
Miyazaki	79.6	78.2	81.0
Kagoshima	79.9	79.4	80.6
Okinawa	85.5	85.6	85.4

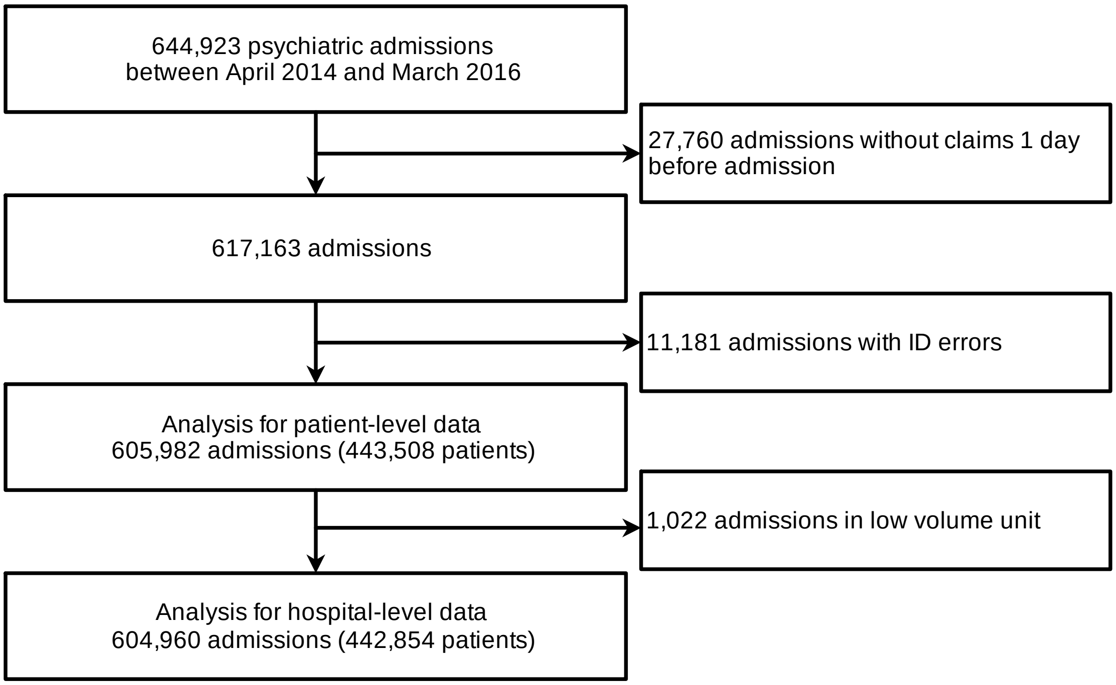

eFigure 1. Flow diagram of included participants

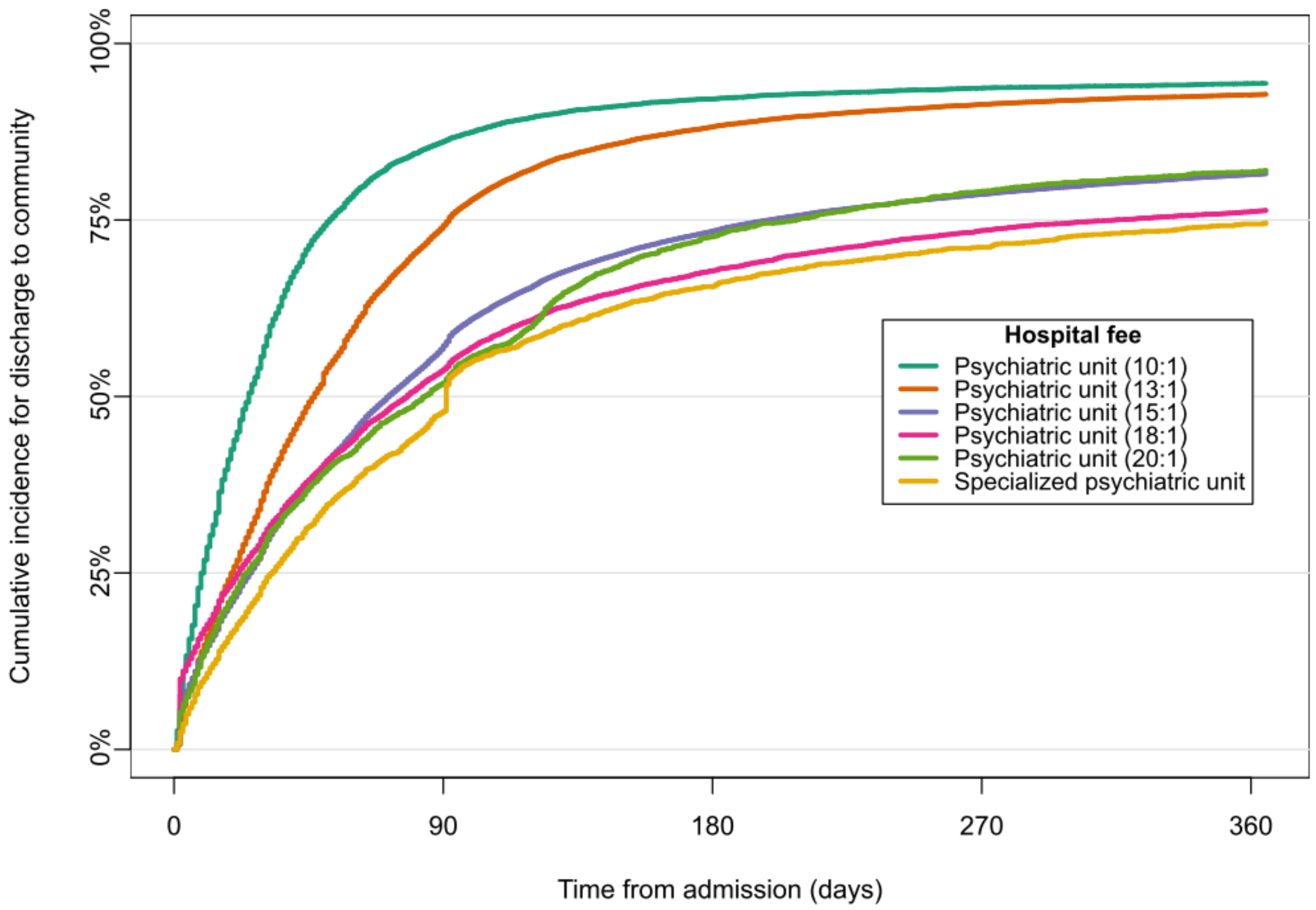

eFigure 2. Cumulative incidence of discharge to community by type of hospital fee (fee-for-service plan)

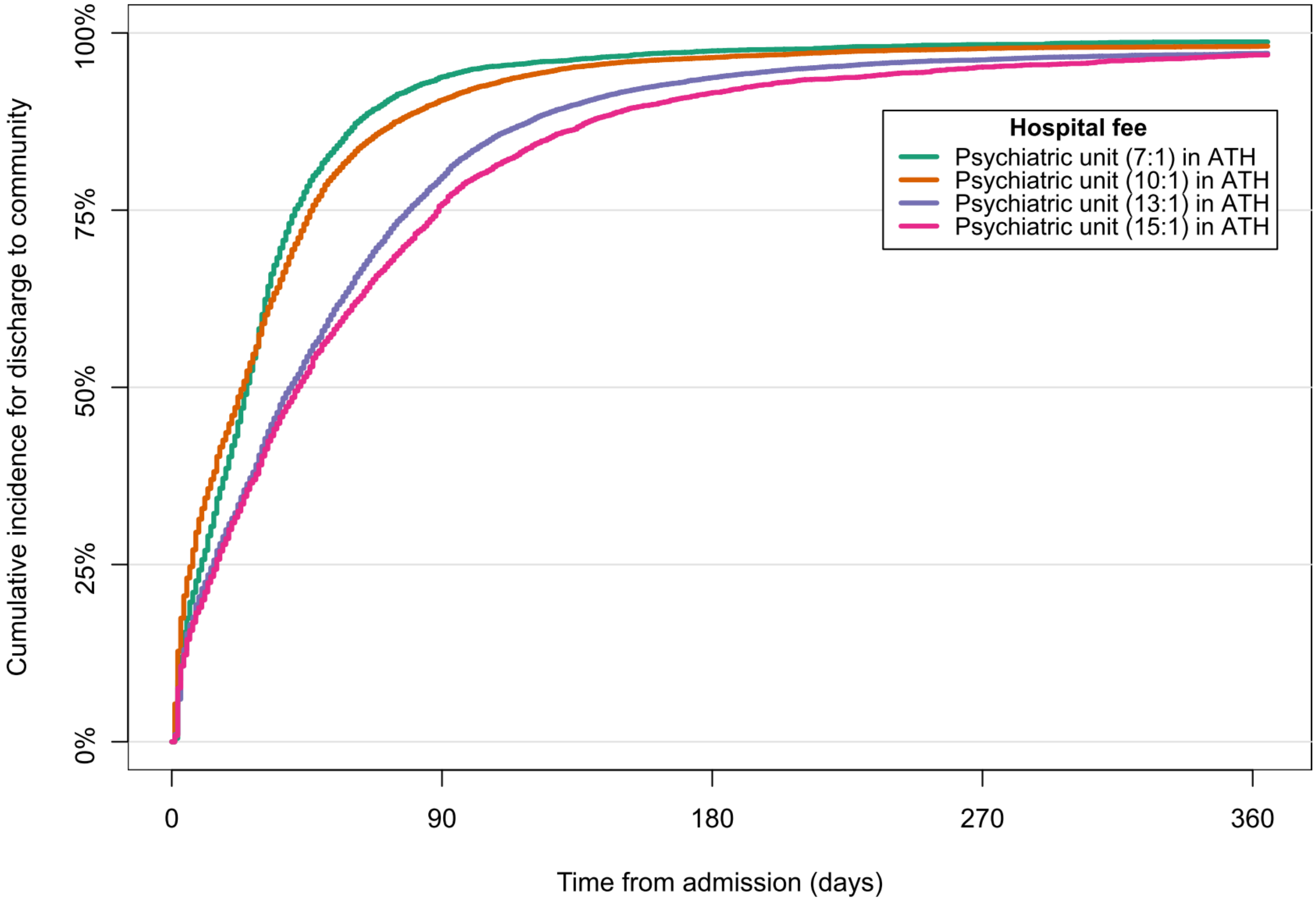

eFigure 3. Cumulative incidence of discharge to community by type of hospital fee (fee-for-service plan in advanced treatment hospitals)

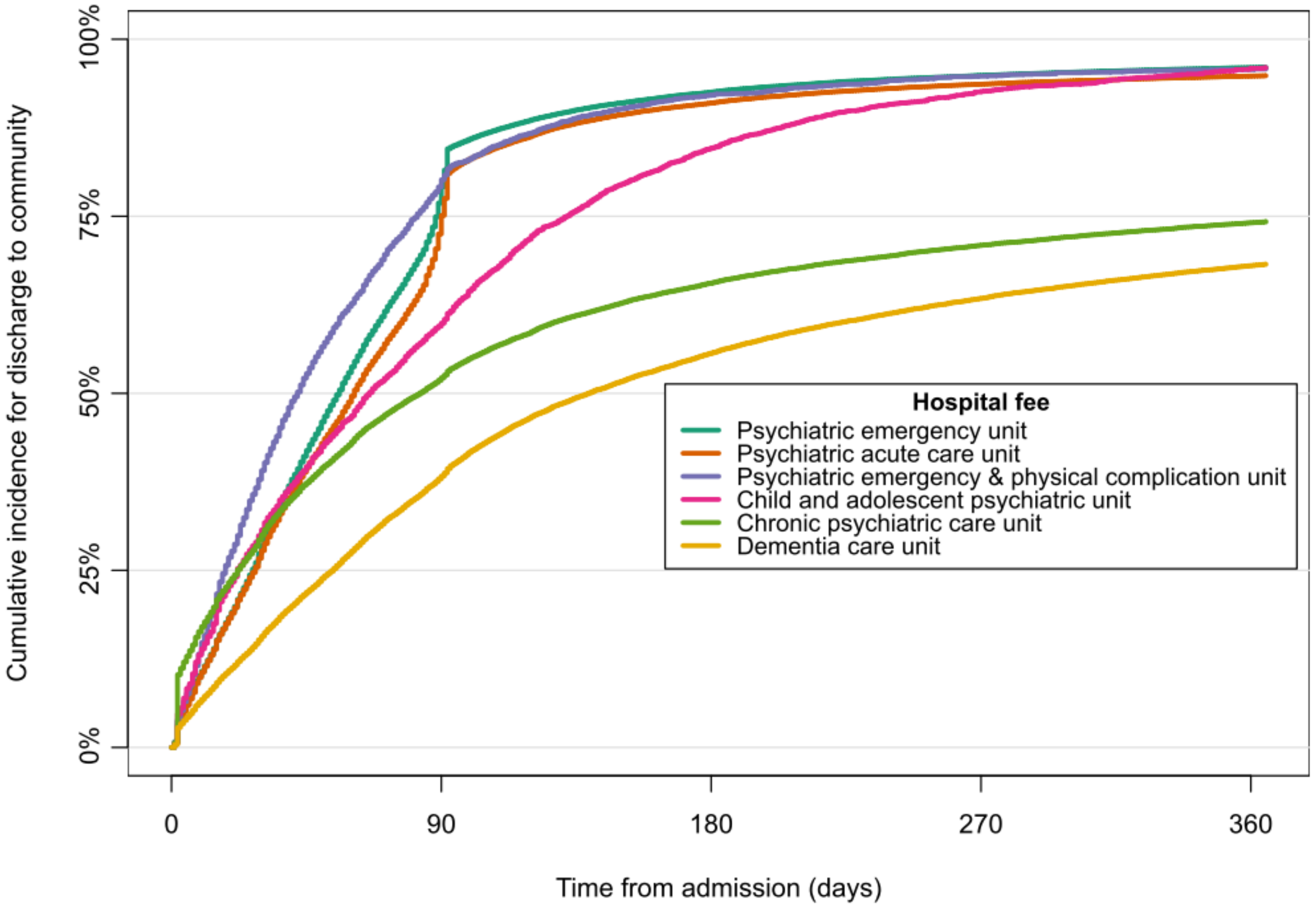

eFigure 4. Cumulative incidence of discharge to community by type of hospital fee (per-diem payment plan)
